# Mediterranean Edible Plants: An Assessment of Their Antioxidant, Radical Scavenger Properties and Their Use as Super Foods, Nutraceuticals, Functional Foods

**DOI:** 10.3390/antiox10050766

**Published:** 2021-05-12

**Authors:** Azzurra Stefanucci, Adriano Mollica

**Affiliations:** Department of Pharmacy, University “G. d’Annunzio” Chieti, Via dei Vestini 31, 66100 Chieti, Italy; a.stefanucci@unich.it

The Mediterranean diet comprises a set of foods that commonly feature in the diet of inhabitants from countries bordering the Mediterranean Sea. This is mainly composed of vegetables, fruit, cereals and legumes—the roles of which have been recognized in the prevention of obesity and chronic degenerative diseases, such as diabetes, tumors, hypertension—thus, it represents a healthy model to follow, since a balanced diet combined with a correct lifestyle is the essential basis for our health [1,2,3,4,5,6,7,8]. Pomegranate (*Punica granatum* L.) juice extract is able to inhibit reactive oxygen species release, increasing the cellular antioxidant response, but also a combination of olive leaf and fruit extracts may be useful to safely reduce hypertension and MetS markers [2,5,6].

Natural bioactive compounds from natural plants, such as *Citrus* flavanones, exhibit antioxidant and anti-inflammatory activities in vitro in a synergistic way; in addition, Tuscan bee pollen is a rich source of essential nutrients and potential nutraceutical product [3]. The origin of the Mediterranean diet is traced back to about 10,000 years ago in the Middle East where wheat, olive oil and wine made up a large part of the crops of this land, to which later, thanks to commercial exchanges, other plants and vegetables were added.

Key American biologists, after careful research, found the low incidence of cardiovascular diseases in the countries of the Mediterranean area. Around the 1950s, the first study in the nutritional field was officially launched. The results were very surprising, showing that the mortality-dependent ischemic heart disease was much lower in the Mediterranean than in other countries. This is due to a diet characterized by a majority of vegetable products exerting a protective function against many chronic degenerative diseases. Unfortunately, this lifestyle and diet are gradually disappearing with alarming and dramatic consequences, especially as regards malnutrition, a biological condition that occurs when energy and nutrients are lacking or excessive compared to individual needs [9].

Expecting a high consumption of cereals, fruit, vegetables and legumes, the Mediterranean diet requires a much less intensive use of natural resources (soil, water) and greenhouse gas emissions than the model based on the consumption of meat and animal fats [10]. *Vitis vinifera* L. grape skin and seeds are clear examples of waste products that are useful as an energy reserve [11,12].

Mixtures of *Citrus sinensis* and *Vitis vinifera* L. cv. *Aglianico N.*, two typical fruits of the Mediterranean diet, possess bioactive polyphenols that protect cardiomyocytes against doxorubicin-induced oxidative stress [13].

The Mediterranean diet involves the consumption of food whilst respecting the rhythms of nature [13,14,15,16,17]. This translates into a reduction in greenhouse crops and related environmental impact, as well as supply and transport costs from distant countries. A “green” solvent-free industrial process was described for a grape seed extract preparation from selected seeds of Veneto region wineries, in the northeast of Italy, by water and selective tangential flow filtration at different porosities [12]. It respects the territory and biodiversity, through different sowing in each area and crop rotation, in order to also guarantee food security. Food diversity and protection of natural areas are crucial to safeguard the world’s threatened species. The Medes Islands in the northwest Mediterranean Sea are a protected natural reserve where wild olive trees are active parts of the vegetation of the Meda Gran island [8,11].

For all these reasons, plant species endowed with various properties, such as anti-inflammatory, anti-aging, antibacterial, and antidiabetic, deserve further investigation and justify the search for new sources of natural antioxidants.
